# Can we still do something-and what? - for a seemingly missing syndrome?: *“Yes we can”*

**DOI:** 10.1186/s13052-019-0735-6

**Published:** 2019-10-29

**Authors:** Raffaele Piumelli, Cinzia Arzilli, Niccolò Nassi, Marta Peruzzi, Carola-Maria Ernst, Cristina Salvatori

**Affiliations:** 1 Sleep Breathing Disorders and SIDS Center, Meyer Children’s Hospital, Viale Pieraccini, 24 50139 Florence, Italy; 2 Department of Neuroscience, Psychology, Drug Research and Child Health, University of Florence, Florence, Italy

**Keywords:** SIDS, SUDI, Diagnostic protocols, Incidence

## Abstract

In this letter, the authors compare the incidence of SUDI and SIDS in the Tuscany Region to the incidence reported by Campi and Bonati in their paper “Can we still do something-and what?- for a seemingly missing syndrome?” that was recently published in this journal. The Tuscany data are directly gathered from the autopsies while the others from the death certificates that are often not reilable, thus causing an understimation of the phenomenon. The real picture of the extent of SIDS is crucial to evaluate the effectiveness of back to sleep campaigns.

Dear Editor,

We have read with interest the paper by Campi and Bonati which first provides the national data about the incidence of the Sudden Infant Death Syndrome (SIDS) and Sudden Unexpected Death in Infancy (SUDI) [1]. The Authors gathered data from the death certificates between 1996 and 2015. SIDS is the “sudden death of an infant which is unexpected in relation to the infant’s history and in which a thorough post-mortem examination fails to demonstrate an adequate cause of death” [2]. Thus, the diagnosis of SIDS is an exclusion diagnosis for which a complete post-mortem is required. In Italy the execution of autopsies is regulated by the decree “Diagnostic protocols in cases of sudden infantile death and unexpected death of the foetus” (14A08847) (GU General Series no. Two hundred seventy-two of 22-11-2014 – Ordinary Supplement no. 89). The unanimous approval of the decree by the Italian Regions Committee has not yet been obtained, therefore its adoption has not yet come into force. Consequently, autopsies are not performed in all cases of sudden unexpected deaths, apart from which, in autopsied cases the lack of standardised procedures may lead to misinterpretations and diagnostic discrepancies, making the data obtained from death certificates unreliable, as is reported in the literature [3, 4]. To overcome this limitation, in the Tuscany Region in 2009 a project aimed at implementing the multiagency management of SUDI cases was initiated in line with the Decree n° 1164/14-12-2009. The aim was to create an integrated organisation working for the benefit of families and professionals to ensure sensitive investigations for identifying the causes of death and providing peer support for bereaved families. From January 2009 to December 2018, 61 SUDI deaths occurred in the Tuscany Region, 30 of which were attributed to SIDS. Autopsies were performed in 85.2% of cases by three groups of selected pathologists. Interestingly, autopsies have been performed in 96% of cases over the last 4 years, demonstrating an increase in the efficacy of the system.

Our data show an incidence of 0.1‰ of SIDS which is more than twofold that reported in the article and even lower than that occurring in other countries in which the phenomenon is accurately monitored and the “back to sleep” campaigns are actively carried out [5]. Unfortunately, in Italy an organised ‘reduce the risk’ campaign is only performed in Tuscany, therefore an even higher SIDS incidence could be expected in other Italian Regions.

Our answer to the Authors’ question: “Can we still do something-and, what? “is therefore: “Yes we can”, but in order not to “miss the syndrome” we must have the real picture of the extent of SIDS through a correct diagnosis.

## Data Availability

Unpublished experimental data are not included in this review.

## Abbreviations

GU: General Series
SIDS: Sudden Infant Death Syndrome
SUDI: Sudden Unexpected Death in Infancy

## References

[1] Campi R, Bonati M (2019). Can we still do something-and what?-for a seemingly missing syndrome?. Ital J Pediatr.

[2] Willinger M, James LS, Catz C (1991). Defining the sudden infant death syndrome (SIDS): deliberations of an expert panel convened by the National Institute of Child Health and Human Development. Pediatr Pathol.

[3] Limerick SR, Bacon CJ (2004). Terminology used by pathologists in reporting on sudden infant deaths. J Clin Pathol.

[4] Corey TS, Hanzlick R, Howard J, Nelson C, Krous H (2007). A functional approach to sudden unexplained infant deaths. Am J Forensic Med Pathol.

[5] Hakeem GF, Oddy L, Holcroft CA, Abenhaim HA (2015). Incidence and determinants of sudden infant death syndrome: a population-based study on 37 million births. World J Pediatr.

